# Supplementary material for: Construction and validation of a gene expression classifier to predict immunotherapy response in primary triple-negative breast cancer
Source: Commun Med (Lond). 2023 Jul 10;3:93. doi: 10.1038/s43856-023-00311-y (PMC10333210; doi:10.1038/s43856-023-00311-y)
Supplement: Supplementary file 2 — Supplementary Information [file 43856_2023_311_MOESM2_ESM.pdf]

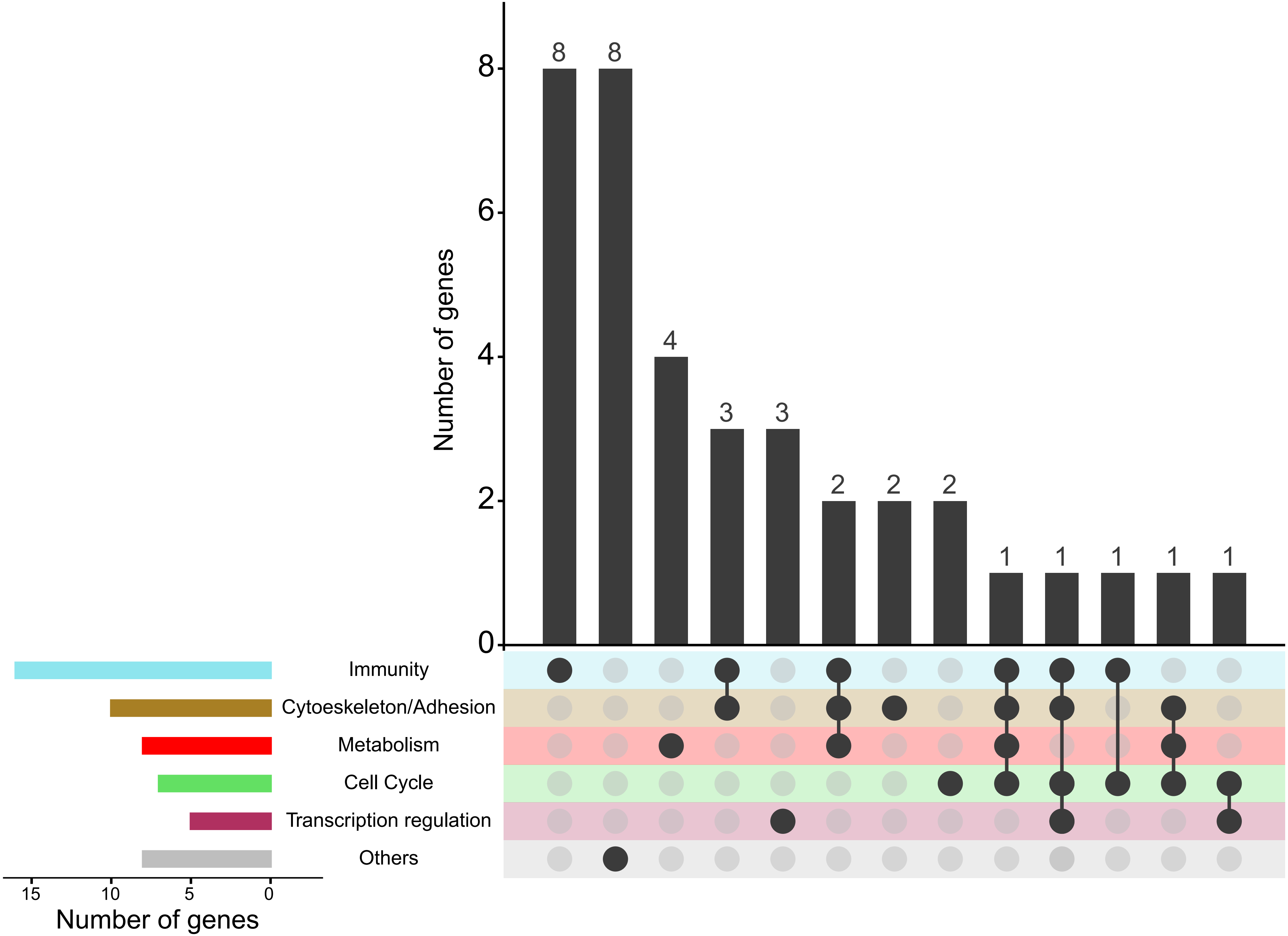

Supplementary Figure 1: UpSet plot representing the number of genes in the classifier involved in different gene functions.

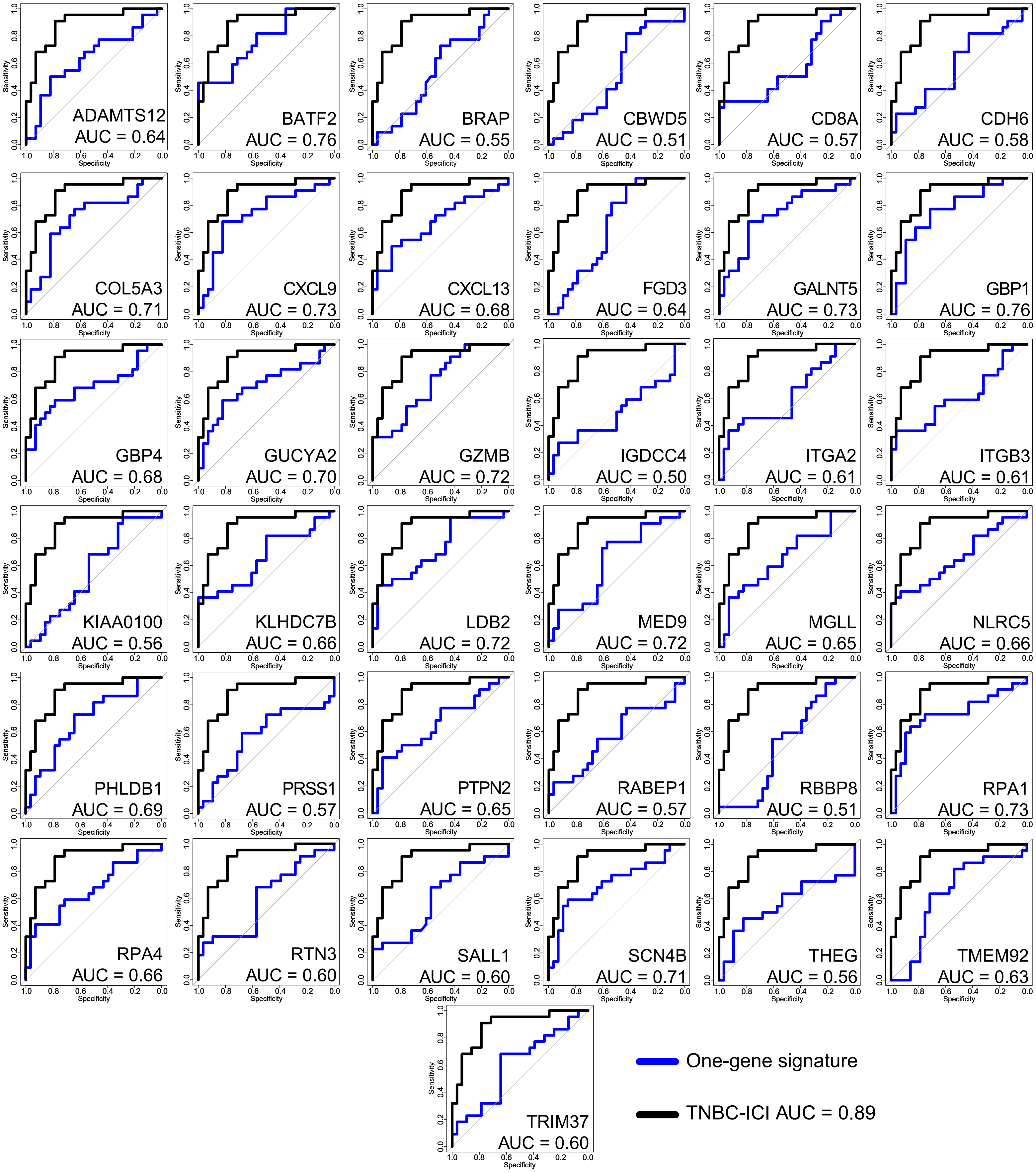

Supplementary Figure 2: ROC Curves displaying the accuracy of each gene in the classifier compared with the accuracy of TNBC-ICI (n=50).

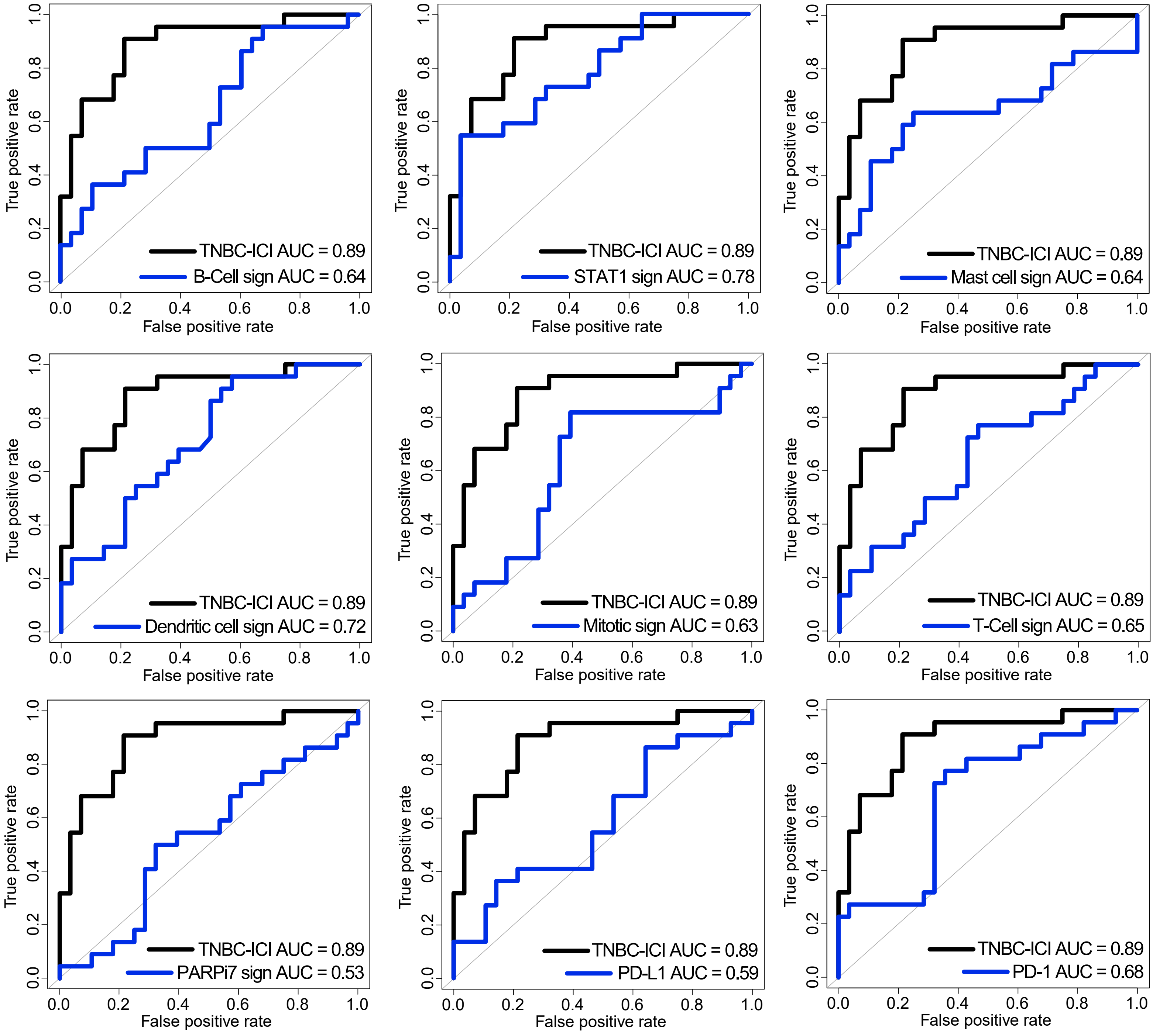

Supplementary Figure 3: ROC Curves representing the performance of various molecular signatures and TNBC-ICI in TNBC (n=50). The machine learning-based signature has higher accuracy than any other molecular signature.

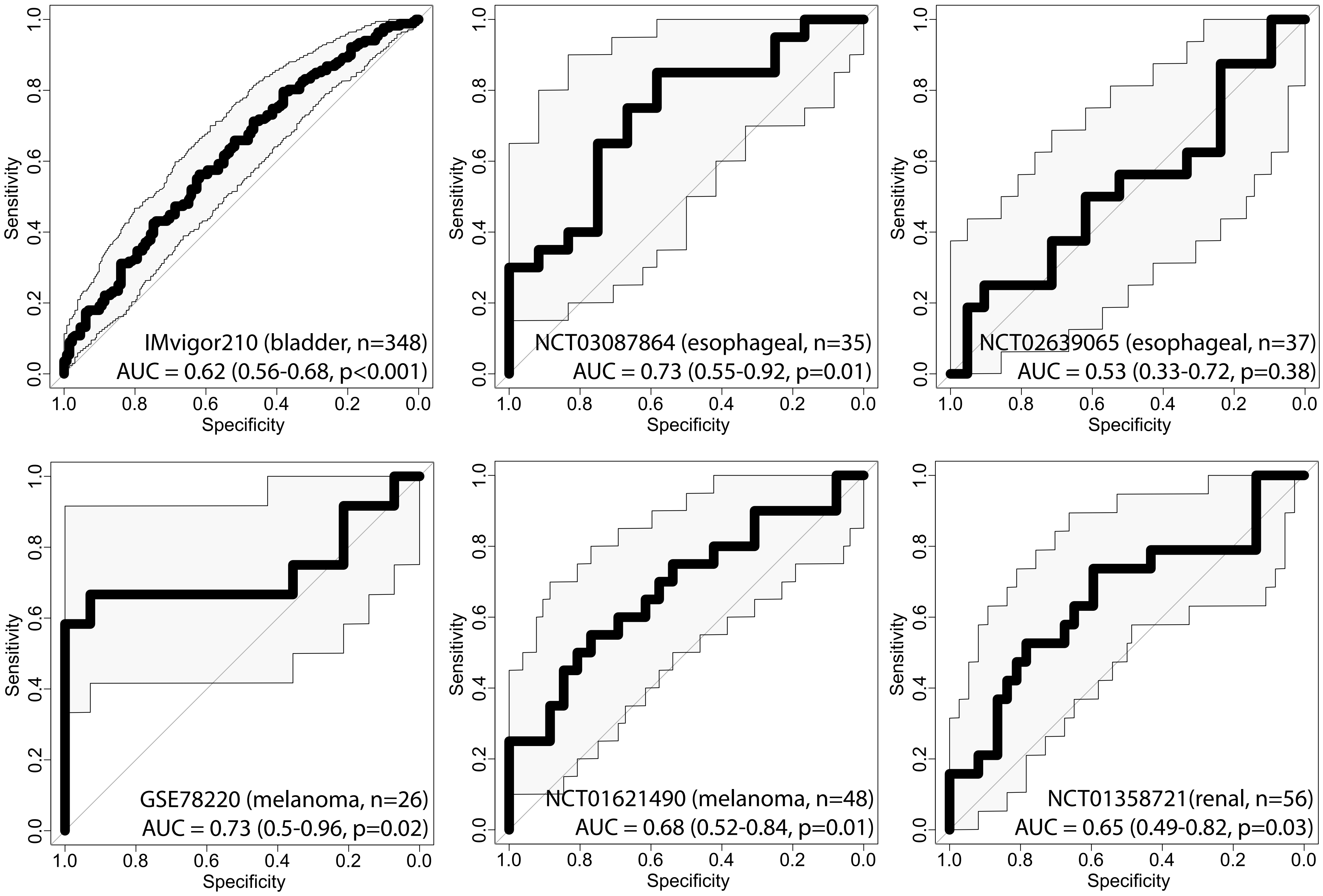

Supplementary Figure 4: ROC Curves displaying the performance of the classifier in different cohorts of non-breast cancer immune checkpoint inhibitors (ICI)-treated patients. From left to right, immune cold tumors (upper panel): bladder (n=348), esophageal (n=35), and esophageal (n=37); immune hot tumors (lower panel): melanoma (n=26), melanoma (n=48) and renal (n=56). The shaded area represents the confidence interval with a 95% confidence level.

Supplementary Table 1: List of the ICI-based studies employed for the construction of the TNBC-ICI classifier

Clinical Trial Name	Phase	Clinical Trial ID(s)	Cancer	Stage	Treatment	N	Data	Access Code
I-SPY2	2	NCT01042379	HR+/HER2- BC	Early	Durvalumab + Olaparib	50	Agendia32627	GSE173839
I-SPY2	2	NCT01042380	HR+/HER2- BC	Early	Pembrolizumab	40	Agendia32627	GSE194040
I-SPY2	2	NCT01042381	TNBC	Early	Durvalumab + Olaparib	21	Agendia32627	GSE173839
I-SPY2	2	NCT01042382	TNBC	Early	Pembrolizumab	29	Agendia32627	GSE194040
IMvigor210	2	NCT02108652 NCT02951767	Bladder	Metastatic	Atezolizumab	348	RNA-seq	IMvigor210
KEYNOTE-052	2	NCT02335424	Bladder	Metastatic	Pembrolizumab	11	Array HTA 2.0	GSE111636
MEDI4736	2	NCT02639065	Esophageal	Locally Advanced	Durvalumab	37	RNA-seq	GSE183924
PERFECT	2	NCT03087864	Esophageal	Early	Atezolizumab	35	RNA-seq	GSE165252
PH1	1	NCT01621490	Melanoma	Unresectable or metastatic	Nivolumab and Nivolumab + Ipilimumab	48	Array U219	E-MTAB-4030
NA	NA	NA	Melanoma	Metastatic	Pembrolizumab	26	RNA-seq	GSE78220
BMS-936558	2	NCT01354431	Renal	Metastatic	Nivolumab	11	Illumina HumanHT-12	GSE67501
BMS-936558	1	NCT01358721	Renal	Metastatic	Nivolumab	56	Array U219	E-MTAB-3218
NA	NA	NA	Thymic carcinoma	Advanced	Pembrolizumab	9	RNA-seq	GSE181815

Supplementary Table 1: Data table indicating the list of the immunotherapy clinical studies used to create and validate the gene expression-based classifiers.

Supplementary Table 2: Genes included in the TNBC-ICI classifier

Gene	Score	Gene Accession	Gene Name	Molecular Function
SALL1	-41.06	NM_001127892	Spalt Like Transcription Factor 1	Organogenesis
GALNT5	-40.09	NM_001329868	Polypeptide N-Acetylgalactosaminyltransferase 5	Protein metabolism
CDH6	-39.48	NM_001362435	Cadherin 6	Cell adhesion
GUCY1A2	-34.01	NM_000855	Guanylate Cyclase 1 Soluble Subunit Alpha 2	Nitric oxide-mediated signaling
ADAMTS12	-30.96	NM_001324511	ADAM Metallopeptidase With Thrombospondin Type 1 Motif 12	Cell adhesion
TMEM92	-28.17	NM_001168215	Transmembrane Protein 92	Immune system
ITGB3	-28.12	NM_000212	Integrin Subunit Beta 3	Cell-surface receptor
PHLDB1	-27.32	NM_001144758	Pleckstrin Homology Like Domain Family B Member 1	Regulation of EMT
RPA4	-26.79	NM_013347	Replication Protein A4	Response to DNA damage
MGLL	-25.96	NM_001003794	Monoglyceride Lipase	Cell migration and invasion
IGDCC4	-25.82	NM_020962	Immunoglobulin Superfamily DCC Subclass Member 4	Immunoglobulin
COL5A3	-24.88	NM_015719.4	Collagen Type V Alpha 3 Chain	Fibrilar collagen
LDB2	-24.63	NM_001130834	LIM Domain Binding 2	Transcription regulation
SCN4B	-24.54	NM_001142348	Sodium Voltage-Gated Channel Beta Subunit 4	Action potential initiation and propagation
PRSS1	-24.24	NM_002769	Serine Protease 1	Tripsine formation
THEG	-23.55	NM_016585	Theg Spermatid Protein	Protein metabolism
ITGA2	-22.38	NM_002203	Integrin Subunit Alpha 2	Receptor for collagens and related proteins
NLRC5	22.37	NM_001330552	NLR Family CARD Domain Containing 5	Innate immune system
GZMB	22.41	NM_001346011	Granzyme B	Immune system
CD8A	22.95	NM_001145873	CD8a Molecule	Immune system
C6ORF89	23.01	NM_001286635	Chromosome 6 Open Reading Frame 89	Cell cycle
RPA1	23.85	NM_001355120	Replication Protein A1	Response to DNA damage
RBBP8	24.63	NM_002894	RB Binding Protein 8, Endonuclease	Cell proliferation
RABEP1	24.66	NM_001083585	Rabaptin, RAB GTPase Binding Effector Protein 1	Vesicle-mediated transport
BATF2	25.75	NM_001300807	Basic Leucine Zipper ATF-Like Transcription Factor 2	Transcription regulation
FGD3	26.26	NM_001083536	FYVE, RhoGEF And PH Domain Containing 3	Cytoskeleton
CXCL13	26.33	NM_001371558	C-X-C Motif Chemokine Ligand 13	Immune system
MED9	26.42	NM_018019	Mediator Complex Subunit 9	Activation of RNA-pol II
BLTP2	26.75	NM_001321560	Bridge-Like Lipid Transfer Protein Family Member 2	Membrane trafficking
PTPN2	27.14	NM_001207013	Protein Tyrosine Phosphatase Non-Receptor Type 2	Multipathway regulator
KLHDC7B	27.50	NM_138433	Kelch Domain Containing 7B	Apoptosis control
ZNG1E	28.39	NM_001024916	Zn Regulated GTPase Metalloprotein Activator 1E	ATP binding activity
CXCL9	28.95	NM_002416	C-X-C Motif Chemokine Ligand 9	Immune system
GBP4	30.10	NM_052941	Guanylate Binding Protein 4	Interferon gamma signaling
RTN3	35.01	NM_001265589	Reticulon 3	BCL-2 translocation
GBP1	35.06	NM_002053	Guanylate Binding Protein 1	Interferon gamma signaling
TRIM37	37.01	NM_001005207	Tripartite Motif Containing 37	Innate immune system

Supplementary Table 2: Table displaying the list of genes in the classifier and their function.
