# Supplementary material for: Construction and validation of a gene expression classifier to predict immunotherapy response in primary triple-negative breast cancer
Source: Commun Med (Lond). 2023 Jul 10;3:93. doi: 10.1038/s43856-023-00311-y (PMC10333210; doi:10.1038/s43856-023-00311-y)
Supplement: Supplementary file 3 — Description of Additional Supplementary Files [file 43856_2023_311_MOESM3_ESM.pdf]

Description of Additional Supplementary File

File Name: Supplementary Data 1

Description: Data table containing the 500 most differentially expressed genes between immune checkpoint inhibitors (ICI) plus chemotherapy responder and non-responder patients pan-cancer. These genes were employed as the initial input in the random forest analysis.
