# Supplementary material for: Construction and validation of a gene expression classifier to predict immunotherapy response in primary triple-negative breast cancer
Source: Commun Med (Lond). 2023 Jul 10;3:93. doi: 10.1038/s43856-023-00311-y (PMC10333210; doi:10.1038/s43856-023-00311-y)
Supplement: Supplementary file 5 — Reporting Summary [file 43856_2023_311_MOESM5_ESM.pdf]

nature portfolio

Corresponding author(s): Diego Marzese

Last updated by author(s): 2023-05-30

Reporting Summary

Nature Portfolio wishes to improve the reproducibility of the work that we publish. This form provides structure for consistency and transparency in reporting. For further information on Nature Portfolio policies, see our Editorial Policies and the Editorial Policy Checklist.

Statistics

For all statistical analyses, confirm that the following items are present in the figure legend, table legend, main text, or Methods section.

n/a	Confirmed	
☐	☒	The exact sample size (*n*) for each experimental group/condition, given as a discrete number and unit of measurement
☒	☐	A statement on whether measurements were taken from distinct samples or whether the same sample was measured repeatedly
☐	☒	The statistical test(s) used AND whether they are one- or two-sided<br>*Only common tests should be described solely by name; describe more complex techniques in the Methods section.*
☐	☒	A description of all covariates tested
☐	☒	A description of any assumptions or corrections, such as tests of normality and adjustment for multiple comparisons
☐	☒	A full description of the statistical parameters including central tendency (e.g. means) or other basic estimates (e.g. regression coefficient) AND variation (e.g. standard deviation) or associated estimates of uncertainty (e.g. confidence intervals)
☐	☒	For null hypothesis testing, the test statistic (e.g. *F*, *t*, *r*) with confidence intervals, effect sizes, degrees of freedom and *P* value noted<br>*Give P values as exact values whenever suitable.*
☒	☐	For Bayesian analysis, information on the choice of priors and Markov chain Monte Carlo settings
☐	☒	For hierarchical and complex designs, identification of the appropriate level for tests and full reporting of outcomes
☒	☐	Estimates of effect sizes (e.g. Cohen's *d*, Pearson's *r*), indicating how they were calculated

Our web collection on statistics for biologists contains articles on many of the points above.

Software and code

Policy information about availability of computer code

Data collection	All employed data was obtained from publicly available databases.
Data analysis	All the code employed in the study is available at https://github.com/mensenyat/TNBC-ICI and at the Zenodo repository. These files include a simplified version of the code that can be employed to check the validity of the method and a dataset and code that can be used to apply TNBC-ICI to new samples. This code will be updated in GitHub with data from additional public datasets when they are released.

For manuscripts utilizing custom algorithms or software that are central to the research but not yet described in published literature, software must be made available to editors and reviewers. We strongly encourage code deposition in a community repository (e.g. GitHub). See the Nature Portfolio guidelines for submitting code & software for further information.

Data

Policy information about availability of data

All manuscripts must include a data availability statement. This statement should provide the following information, where applicable:

- Accession codes, unique identifiers, or web links for publicly available datasets
- A description of any restrictions on data availability
- For clinical datasets or third party data, please ensure that the statement adheres to our policy

All the data employed in the study has been obtained from publicly available databases, including the Gene Expression Omnibus (GEO), ArrayExpress, and directly from the IMvigor210CoreBiologies R package. All the accession numbers are available in Supplementary Table 1. Source data for the figures are available at the Zenodo repository.

Human research participants

Policy information about studies involving human research participants and Sex and Gender in Research.

Reporting on sex and gender	Most samples were labeled as females due to the characteristics of the studied cancers (Breast Cancer was the main studied cancer).
Population characteristics	Various cohorts of cancer patients were employed in the analysis. The two main cohorts represented 50 patients with primary triple-negative breast cancer treated with immune checkpoint inhibitors. Additional cohorts represented a total of 909 cancer patients, treated or not treated with immune checkpoint inhibitors.
Recruitment	All data was obtained from publicly available databases and the patients were recruited in their host institutions.
Ethics oversight	The data included in all publicly available cohorts were collected according to the respective institutional review board approvals following the human subjects protection and data access policies. Written consent was obtained from all patients. All samples were deidentified and coded following the Health Insurance Portability and Accountability Act (HIPAA) guidelines.

Note that full information on the approval of the study protocol must also be provided in the manuscript.

Field-specific reporting

Please select the one below that is the best fit for your research. If you are not sure, read the appropriate sections before making your selection.

☒ Life sciences ☐ Behavioural & social sciences ☐ Ecological, evolutionary & environmental sciences

For a reference copy of the document with all sections, see nature.com/documents/nr-reporting-summary-flat.pdf

Life sciences study design

All studies must disclose on these points even when the disclosure is negative.

Sample size	Gene expression data from 909 patients were used in total. For the generation of the classifier, 31 TNBC patients were used as the training cohort and 19 were used as the first validation cohort. Then, the rest of the patients were used as secondary validation cohorts.
Data exclusions	Patients without response and relapse data and patients with low data quality were removed from further analysis.
Replication	The process for the selection of the signatures was iterated 1,000 times to increase the reproducibility of the method.
Randomization	The initial TNBC cohort was split randomly in a 60% Training cohort and a 40% Validation cohort.
Blinding	The study was not blinded due to the characteristics of the analysis, requiring homogeneity between training and validation cohorts. Furthermore, the data was obtained in previous studies and was not affected by the analysis.

Reporting for specific materials, systems and methods

We require information from authors about some types of materials, experimental systems and methods used in many studies. Here, indicate whether each material, system or method listed is relevant to your study. If you are not sure if a list item applies to your research, read the appropriate section before selecting a response.

Materials & experimental systems

n/a	Involved in the study	
☒	☐	Antibodies
☒	☐	Eukaryotic cell lines
☒	☐	Palaeontology and archaeology
☒	☐	Animals and other organisms
☐	☒	Clinical data
☒	☐	Dual use research of concern

Methods

n/a	Involved in the study	
☒	☐	ChIP-seq
☒	☐	Flow cytometry
☒	☐	MRI-based neuroimaging

Clinical data

Policy information about clinical studies

All manuscripts should comply with the ICMJE guidelines for publication of clinical research and a completed CONSORT checklist must be included with all submissions.

Clinical trial registration	All data was obtained from already published clinical trials.
Study protocol	All data was obtained from already published clinical trials.
Data collection	All data was obtained from already published clinical trials.
Outcomes	All data was obtained from already published clinical trials.
